# TerraGrow: Integrated platform for real time plant monitoring and automated watering system with IoT and fuzzy Sugeno Algorithm

**DOI:** 10.1016/j.ohx.2025.e00724

**Published:** 2025-11-24

**Authors:** Prima Wijayakusuma, Galang Persada Nurani Hakim, Bin Li

**Affiliations:** a Beijing Institute of Technology, China; b Universitas Mercu Buana, Indonesia

**Keywords:** Internet of Things (IoT), Wireless Sensor Networks (WSN), Fuzzy Sugeno control, Smart irrigation, Soil moisture and pH sensing, ESP32

## Abstract

Global water scarcity, climate variability, and rising input costs are pushing agriculture toward precise, evidence-based irrigation, yet many available systems remain proprietary, expensive, and difficult to adapt. TerraGrow is an open-source, low-cost controller that runs all sensing and closed-loop irrigation locally on a single ESP32, using real-time soil moisture, pH, temperature, and humidity inputs with a Sugeno-type fuzzy policy. Its modular and ergonomic hardware with a printable enclosure, integrated pump driver, and labeled connectors lets non-experts assemble, calibrate, and service the unit quickly in the field. Compared with earlier low-cost IoT irrigation nodes, TerraGrow’s novelty lies in fully local multi-sensor fuzzy control and an easy-to-deploy form factor supported by complete and reproducible design files. Bench and greenhouse tests showed stable moisture regulation and reduced unnecessary watering, enabled by precise sensing and a consistent hardware and firmware implementation. By combining local autonomy, simple deployment, and open documentation, TerraGrow makes practical precision irrigation more accessible to resource-constrained growers.

## Graphical abstract

**Figure:**
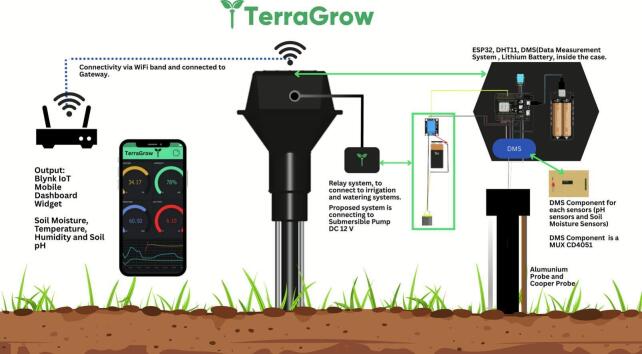

**Specifications table:** 

Hardware name	*TerraGrow*
Subject area	• Engineering and materials science • Environmental, planetary and agricultural sciences
Hardware type	• Electrical engineering and computer science • Measuring physical properties and in-lab sensors • Field measurements and sensors
Closest commercial analog	*No commercial analog is available*
Open source license	*Creative Commons Attribution 4.0 International*
Cost of hardware	*USD 119.00*
Source file repository	*https://doi.org/10.5281/zenodo.16880646*

## 1 Hardware in context

*Agriculture now has to produce more with less water, less energy, and less labor due to climate stress, freshwater scarcity, and rising input costs* [1,2]*. Because of this, irrigation is shifting from manual schedules to closed-loop control: water is applied only when real soil and microclimate measurements indicate that the plant actually needs it* [[3], [4], [5]]*. Prior work in precision irrigation and Agriculture 4.0 shows that this data-driven approach can stabilize root-zone moisture, avoid over-watering, and improve water-use efficiency under heat and drought conditions* [6]*.*

*Recent smart irrigation systems combine sensing, wireless telemetry, and automatic control. Typical research prototypes monitor soil moisture, temperature, humidity, and sometimes pH; send those values to a dashboard; and use fuzzy logic or lightweight decision rules to switch a pump or valve* [7]*. Some platforms even manage multi-hectare orchards using LoRaWAN sensor nodes and a central gateway* [[9], [10], [11]]*. These studies confirm that smart irrigation is technically mature and can save water at scale,*[8,9]*. At the same time, recent work shows that low-cost microcontrollers such as the ESP32 can run fuzzy controllers locally, drive irrigation hardware, and report live status to a phone without needing a remote server* [3,4,8,12,14,15]*. Sugeno-type fuzzy inference is effective because it can turn multiple sensor readings into a direct watering action with very low computation* [3,4,8,12,15]*.*

*However, most reported systems are still difficult for small growers to actually use. They often depend on proprietary cloud services or custom long-range gateways, require higher-cost probes (especially for soil chemistry), and are not built to be installed or serviced by non-experts* [[6], [7], [8], [9], [10], [11]]*. Many platforms only monitor conditions instead of running fully automatic pump control at plot scale* [[9], [10], [11], [12], [13]]*.*

*TerraGrow is designed to close that gap. It combines multi-point soil moisture sensing, air temperature and humidity monitoring, and a low-cost soil pH probe with an ESP32-based Sugeno fuzzy controller that decides when and how long to water, locally and automatically. Its physical layout is intentionally modular and ergonomic: the sensing stack, relay/pump stage, enclosure, and cable routing are arranged so the unit can be placed next to a pot or bed, connected with minimal wiring skill, and serviced by a non-expert. The user interface is a simple phone dashboard for live moisture and pump state.*

*In short, TerraGrow follows the same direction as recent precision irrigation work closed-loop, sensor-based, fuzzy-controlled watering, but focuses on being low-cost, modular, and easy to handle by small-scale growers, not just by large, infrastructure-rich farms.*

## 2 Hardware description

**Fig. 1:**
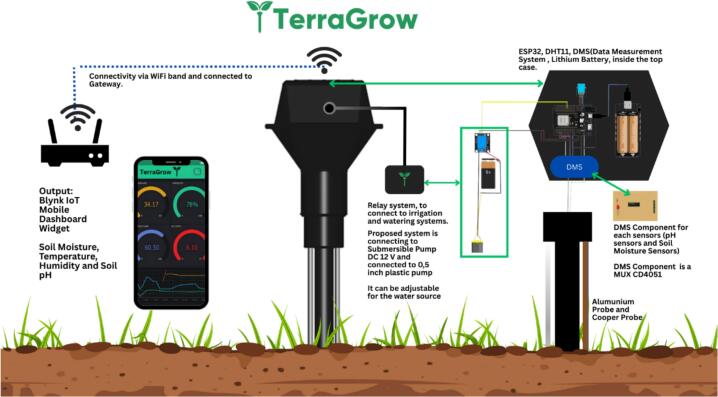
End‑to‑end layout of TerraGrow.

Fig. 1 *illustrates the end‑to‑end layout of TerraGrow, a probe stake integrates the sensing stack and a top‑cap electronics bay that houses the ESP32, the Data Measurement System (DMS) based on a CD4051 analog multiplexer, the DHT11, and a 18,650 lithium battery pack with shield. Multi‑point soil‑moisture electrodes and an aluminum‑electrode pH pair terminate at the DMS, where channels are time‑multiplexed to a single ESP32 ADC line for scalable acquisition. The controller connects via Wi‑Fi to a local gateway/router and publishes telemetry to the Blynk mobile dashboard, which displays soil‑moisture, temperature, humidity, and pH and provides manual override. Irrigation is driven through a relay block to a submersible pump (field builds may use 5–12  V pumps depending on supply), feeding a 0.5‑inch line to the root zone; the source reservoir is user‑configurable. This architecture minimizes I/O usage, centralizes power and signal conditioning in the cap, and keeps electrodes and plumbing at soil level for robust field deployment* [2,10]*.*

*The TerraGrow platform is organized into three major units:*

*(i) Sensing unit — three soil‑moisture electrode pairs (resistive) for multi‑point volumetric‑water‑content inference; DHT11 for ambient T/RH; and an aluminum‑electrode soil‑pH probe;*

*(ii) Processing unit — NodeMCU ESP32 with a CD4051 (8:1) analog MUX feeding a single ADC channel and firmware implementing a Sugeno FIS;*

*(iii) Actuation unit — a 5  V/10 A relay switching a 5  V submersible pump. The electronics are housed in a ventilated PLA* *enclosure.se*.

**Fig. 2:**
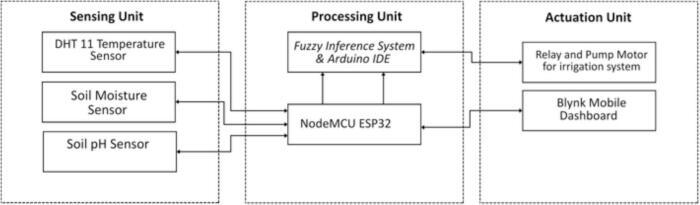
TerraGrow’s architecture diagram systems.

*In* Fig. 2 *presents TerraGrow’s architecture, highlighting the three units sensing, processing, and actuation and the left‑to‑right data flow from sensors to the relay‑driven pump.*

### 2.1 Sensing unit

*The unit measures the key variables that control irrigation: soil moisture, air temperature–humidity, and soil pH. Three electrode points are placed in the root zone to capture spatial variability* [7,18]*. Soil moisture uses paired resistive electrodes wetter soil lowers bulk resistivity and ADC reading, while drier soil raises both. These signals feed the controller in real time* [7,30]*. Air temperature and relative humidity (DHT11) indicate evaporative demand, allowing rules to reduce irrigation in cool–humid and extend it in hot–dry conditions* [2,3]*. The pH channel uses an aluminum-electrode pair read by the Data Measurement System (DMS) and converted to pH via two- or three-point buffer calibration (pH 4/7/10), following standard electrochemical practice* [23,32]*. Physically, the top-cap houses the ESP32 (U1), battery (B1), DHT11 (S2), and two DMS boards with CD4051 multiplexers. The harness exits as S1 (copper moisture electrodes) and S3 (aluminum pH electrodes). Analog sources are time-multiplexed to a single ESP32 ADC line to keep wiring short, reduce noise, and minimize pin count* [6,13]*.*

### 2.2 Soil‑moisture sensor (electrode‑based)

*The soil-moisture channel uses paired copper electrodes placed near the root zone. Current flows through the soil’s and soil increases conductivity and lowers resistance and ADC voltage, while drying raises both. Three sensing points (CH1–CH3) at consistent spacing and depth capture lateral and depth variations from roots and drainage, improving representativenes]. Each electrode pair connects to the DMS and is read sequentially through a CD4051 multiplexer, allowing multiple channels to share one ESP32 ADC pin* [13]*. During setup, raw ADC codes are recorded under “dry” and “field-wet” conditions and mapped to a site-specific moisture scale* [7]*.*

### 2.3 Soil‑pH sensor (aluminum electrodes)

**Fig. 3:**
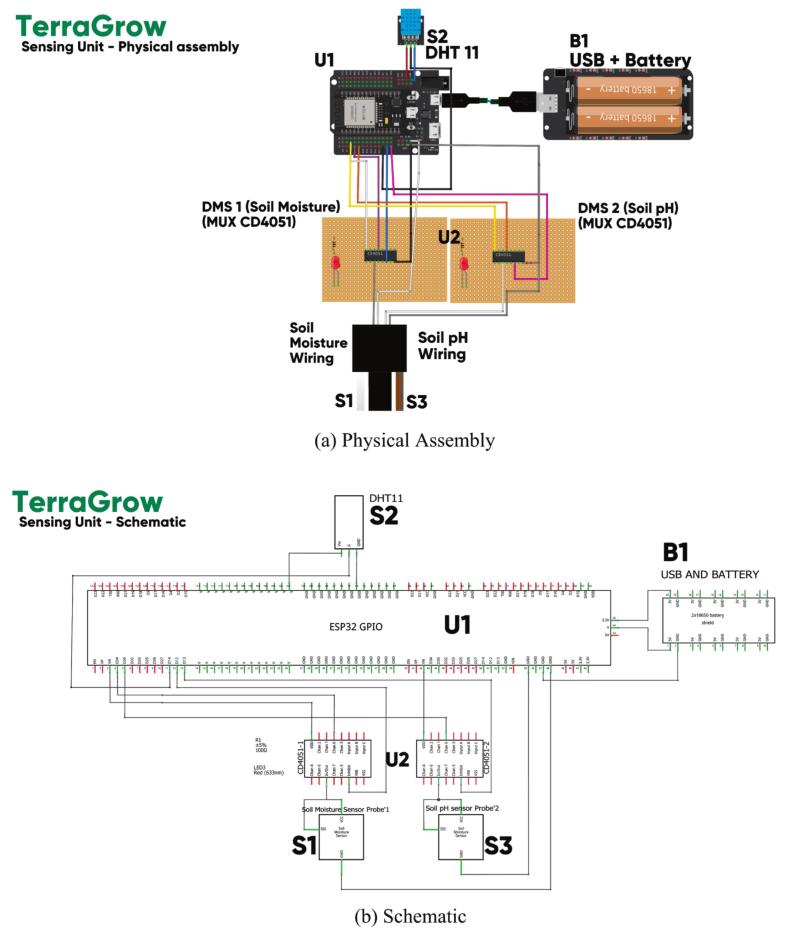
Sensing Unit. (a) Physical assembly and (b) Schematic.

*The soil-pH channel uses an aluminum electrode pair read through the DMS multiplexer. The ESP32 maps ADC code to pH using a simple linear fit after two- or three-point buffer calibration (pH 4/7/10), which is standard for low-cost electrochemical sensing* [23,32,35]*. Live pH is logged along with moisture and RH to flag nutrient or liming stress in real time. As in* Fig. 3(a) *to* Fig. 3(b)*, CH1–CH3 connect to DMS-1 (CD4051) inputs (e.g., Y0–Y2) with the common output returning to a single ESP32 ADC line* [22,23]*.*

### 2.4 Air temperature and humidity sensor (DHT11)

*The ambient channel measures air temperature and relative humidity, key drivers of evapotranspiration and irrigation demand* [2,3]*. A low-cost DHT11 sensor provides a simple digital interface to the ESP32 without analog conditioning* [19]*. The sensor is mounted in a vented, shaded cavity of the top-cap to avoid heating and splash. Readings are sampled every few seconds, filtered with a short moving average, and optionally offset-calibrated. In the fuzzy controller, hot–dry conditions trigger longer irrigation, while cool–humid conditions suppress watering* [3]*.*

*As shown in* Fig. 3(a)*, the ESP32 (U1), DHT11 (S2), battery (B1), and two DMS boards are mounted in the top-cap, with harnesses S1 (moisture) and S3 (pH) exiting separately for easier service. Status LEDs assist setup diagnostics.* Fig. 3(b) *shows the schematic: the ESP32 drives CD4051 select lines (S0–S2), each multiplexer routes one of Y0–Y7 to a single ADC line, and all grounds are common. Only Y0–Y2 are used for moisture channels, leaving spare inputs for expansion* [13]*.*

### 2.5 Processing unit

*The ESP32 (U1) is the main controller. It reads moisture and pH signals through the CD4051 multiplexers, drives the select lines (S0–S2), and reads temperature and humidity from the DHT11 on a single GPIO* [6,12,13]*. Placing the ESP32 in the top-cap keeps analog paths short and improves reliability in the field* [6,13]*. The firmware cycles through each channel (moisture CH1–CH3 and pH), selects it, waits briefly, samples the ADC, filters the reading, and checks for faults before using it in the fuzzy controller* [7,13]*. Calibration from setup (dry / field-wet for moisture, pH 4/7/10 for pH) is applied to convert raw ADC values into real units* [23,25]*. Settings such as Wi-Fi, calibration, and pump safety limits are saved in non-volatile memory so the device can be updated in the field without reflashing, which keeps operation simple for small growers* [2,10]*.*

### 2.6 Fuzzy Sugeno control

*TerraGrow uses a Sugeno-type fuzzy controller that combines three inputs, soil moisture, air temperature, and relative humidity to produce a single output: pump on-time / duty cycle. This approach captures nonlinear crop water demand and runs in real time on a low-cost ESP32. Each input is assigned simple membership sets. Rules follow agronomic logic, such as “IF soil is dry AND hot AND air is low humidity THEN pump longer,” and “IF soil is wet THEN pump off.” Defuzzification uses the standard weighted average form of Sugeno inference* [16]*. Rule weights were tuned so that soil moisture stayed in the 60–80 % target band during tests, and the firmware also enforces maximum on-time, mandatory rest intervals, and duty-cycle limits to avoid over-irrigation and pump overheating. The full rule base and safety limits are included in the released firmware and can be retuned for other crops and condition*s. Table 1 showing *membership sets of fuzzy logic in the systems.*

**Table 1:** Membership sets (initial values; tune per site).

**Variable**	**Linguistic term**	**Range**	**Fuzzy Membership Range**	**Note**
**Soil moisture**	**Low**	**< 35 %**	**Low**	**Below agronomic target; risk of stress**
	**Medium**	**35–60 %**	**Medium**	**Within control band**
	**High**	**> 60 %**	**High**	**Above target; inhibit irrigation**
**Temperature**	**Cold**	**< 22 °C**	**0 0 9 18**	**Low evaporative demand**
	**Good**	**22–30 °C**	**9 18 27 36**	**Typical greenhouse/daytime**
	**Hot**	**> 30 °C**	**27 36 50 50**	**High evaporative demand**
**Humidity**	**Dry**	**< 50 %**	**Low**	**Dry air; faster evaporation**
	**Normal**	**50–70 %**	**Medium**	**Typical range**
	**Moist**	**> 70 %**	**High**	**Damp air; slower evaporation**

*In this design, the fuzzy controller directly outputs the relay command (irrigation level). The relay has three states: Off (no pump, value = 0), Mid (pump on briefly ∼ 5 s, value = 0.5), and On (pump fully active, value = 1). The final command is the weighted average of the membership strengths for these three states and is defined as:*

(1) $$\text{Output}=\frac{\mu_{\text{Off}}\left(x\right)\cdot \text{Off}+\mu_{\text{Mid}}\left(x\right)\cdot \text{Mid}+\mu_{\text{On}}\left(x\right)\cdot \text{On}}{\mu_{\text{Off}}\left(x\right)+\mu_{\text{Mid}}\left(x\right)+\mu_{\text{On}}\left(x\right)}$$

*Off, Mid, and On are the crisp values (0; 0.5; 1), while μ_Off(x), μ_Mid(x), and μ_On(x) are the membership degrees induced by the environmental conditions at time x (temperature, relative humidity, and soil moisture). This weighted‑average form yields a smooth, proportional decision with respect to the available fuzzy evidence. In TerraGrow, the resulting scalar is translated into a watering duration (seconds) and guarded by safety timeouts to prevent over‑irrigation, complementing the fuzzy rules with hard limits* [[14], [15], [16]]*.*

### 2.7 Data Measurement system (DMS)

*The Data Measurement System (DMS) routes multiple analog probes to a single ESP32 ADC input using two CD4051 8:1 analog multiplexers: one for the soil-moisture electrodes and one for the pH electrodes* [13,22]*. The ESP32 drives the select lines (S0–S2) to choose inputs Y0–Y7, waits a short settling delay, samples the ADC on COM, and steps to the next channel. Each channel is then filtered with a short moving average and checked for opens/outliers before being passed to the control layer* [19,22]*. All analog runs are kept short, share a common ground, and use simple shielding to limit noise, pickup, and polarization effects that are typical in low-cost resistive and electrochemical sensing* [7,18,23] *This time-multiplexed readout allows multiple soil points (moisture and pH) to be monitored with minimal wiring and without adding extra ADC pins.*

### 2.8 Actuation unit

**Fig. 4:**
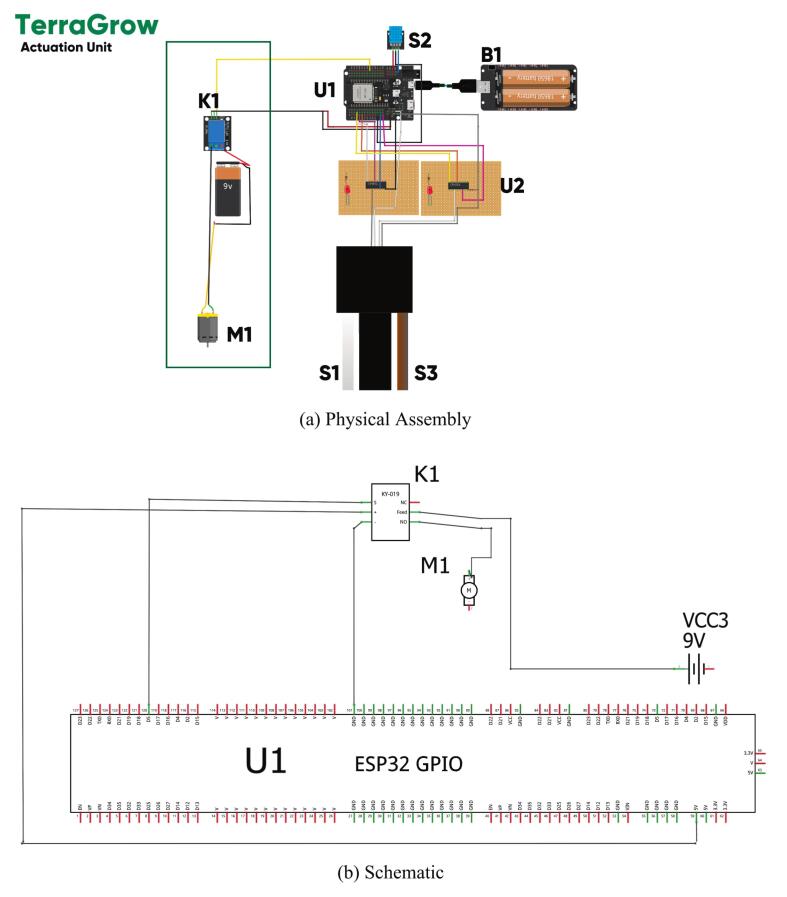
Actuation Unit. (A) shows the physical layout in the top-cap: ESP32 (U1), DMS (U2), DHT11 (S2), relay (K1), battery (B1), and the incoming moisture (S1) and pH (S3) harnesses. (B) shows the wiring schematic ESP32 GPIO drives the relay, the relay switches the pump through the NO contact, and all grounds join at the controller. This keeps high-current lines away from the analog part.

*The actuation stage converts the controller’s command into water flow at the root zone. As shown in* Fig. 4(a) *and* Fig. 4(b)*, the ESP32 (U1) drives a relay module (K1) that switches a submersible DC pump (M1), while the power pack B1 supplies the low‑voltage rails. The relay provides galvanic isolation between the logic domain and the wet‑area pump supply, which is essential for robustness and safety in greenhouse deployments* [5]*. Field builds commonly use a 5–12  V pump matched to the relay contact rating and reservoir head; the wiring follows the NO (normally‑open) path so the default state is off.*

*The top-cap houses the ESP32 (U1), DMS board (U2), DHT11 (S2), relay (K1), and battery pack (B1), with harnesses S1 (moisture) and S3 (pH) extending from the sensing stage. The schematic shows one GPIO driving K1, which switches the pump (M1) through a normally open contact to the external supply. Common grounding and short high-current paths reduce interference. The fuzzy controller output defines pump on-time or duty cycle, and the firmware converts this into timed relay pulses with rest intervals to prevent over-irrigation and pump stress.*

### 2.9 Connectivity & HMI

*The ESP32 reports telemetry to a smartphone dashboard via Wi‑Fi, allowing real‑time visualization of moisture, temperature, humidity, and pH; the interface also offers manual override, parameter tweaks, and notification of alarms, which together reduce the manual workload while keeping the operator in the loop. Historical logs produced by the platform are used during validation to adjust calibration and to refine membership functions and rule weights, letting the control policy adapt to soil type and microclimate without rewriting firmware* [1,4]*.*

### 2.10 Power & enclosure

*A two lithium battery pack 18,650 and a regulated 5 V with enclosure rail supply the controller, the DMS, sensors, and the pump, with fusing and basic protections as implemented in the thesis build to guard against shorts and moisture ingress in the enclosure. The housing is a vented PLA top‑cap designed to shed drips and provide airflow; cable strain relief and gasketed pass‑throughs reduce mechanical stress on electrodes and plumbing and help maintain long‑term durability in greenhouse or small‑plot settings.*

### 2.11 IoT configuration

*The mobile dashboard displays live soil moisture (three channels), air temperature, humidity, pH, and pump state, and includes a manual override for priming or forced irrigation without interrupting closed-loop control* [10,18]*. Each variable is sent on its own virtual channel, with alerts triggered when moisture or pH deviate from target ranges* [17,21]*. Historical logs help refine calibration and fuzzy rules without reflashing. TerraGrow uses Blynk as its IoT layer, enabling Wi-Fi telemetry, remote control, and notifications through Blynk Cloud, consistent with low-cost irrigation system designs* [10,17].

## 3 Design files summary

*The design-file package includes all artifacts required to fully reproduce the TerraGrow system from end to end. Each asset is properly versioned and licensed, and the complete bundle is archived on Zenodo for long-term access and citation at* *https://doi.org/10.5281/zenodo.16880646*, with the individual design assets enumerated in Table 2 and the corresponding hardware components summarized in Table 3.

**Table 2:** Design files summary.

**Design file name**	**File type**	**Open source license**	**Location of the file**
**Illustrations of the end‑to‑end layout and configurations of TerraGrow**	**PDF**	**CC‑BY 4.0**	**https://doi.org/10.5281/zenodo.16880646**
**Electrical schematic**	**PDF**	**CC‑BY 4.0**	**https://doi.org/10.5281/zenodo.16880646**
**Firmware (Arduino)**	**.ino + headers**	**CC‑BY 4.0**	**https://doi.org/10.5281/zenodo.16880646**
**3D‑printed housing Enclosure model**	**.stl,.pdf**	**CC‑BY 4.0**	**https://doi.org/10.5281/zenodo.16880646**
**Supplemental parts**	**.xlsx**	**CC‑BY 4.0**	**https://doi.org/10.5281/zenodo.16880646**

**Table 3:** Bills of material.

**Designator**	**Component**	**Qty**	**Cost per unit** **(USD)**	**Total cost (USD)**	**Source of materials**	**Material type**
**U1**	**NodeMCU ESP32 (Wi–Fi MCU) + GPIO Breakout Board**	**1**	**9.00**	**9.00**	**Shopee**	**Electronic**
**U2**	**CD4051 analog multiplexer (8:1)**	**2**	**1.00**	**2.00**	**Shopee**	**Electronic**
**S1**	**Soil–moisture electrode pair (resistive)**	**1**	**2.00**	**2.00**	**Shopee**	**Electronic**
**S2**	**DHT11 temperature–humidity sensor**	**1**	**3.00**	**3.00**	**Shopee**	**Electronic**
**S3**	**Soil pH probe (aluminum electrodes)**	**1**	**5.00**	**5.00**	**Shopee**	**Electronic**
**K1**	**Relay module, 5 V 10 A**	**1**	**2.50**	**2.50**	**Shopee**	**Electronic**
**M1**	**Submersible water pump, 5 V**	**1**	**10.00**	**10.00**	**Shopee**	**Electronic**
**B1**	**Lithium battery 18650 + protection shield**	**1**	**15.00**	**15.00**	**Shopee**	**Electronic**
**W1**	**Wiring harness & connectors**	**1**	**3.00**	**3.00**	**Shopee**	**Other**
**H1**	**PVC hose 1/4″ (≈1 m)**	**1**	**1.00**	**1.00**	**Shopee**	**Other**
**E1**	**PLA enclosure (3D–printed, vented)**	**1**	**8.00**	**8.00**	**In–house 3D print**	**Polymer**
**P1**	**Passives kit (100 Ω resistors, LED, small protoboard 3 × 3 cm)**	**1**	**2.50**	**2.50**	**Shopee**	**Semiconductor**
**HC1**	**Mini hose clamps for 1/4″ hose (pack of 4)**	**1**	**1.5**	**1.5**	**Shopee**	**Other**
**V1**	**Inline check valve 1/4″**	**1**	**2**	**2**	**Shopee**	**Other**
**JW1**	**Dupont jumper wires F-M, 20 cm, 2.54 mm, 26**–**28 AWG (40 pcs)**	**1**	**2**	**2**	**Shopee**	**Other**
**JW2**	**Dupont jumper wires M−M, 20 cm, 2.54 mm, 26**–**28 AWG (40 pcs)**	**1**	**2**	**2**	**Shopee**	**Other**
**JW3**	**Dupont jumper wires F-F, 20 cm, 2.54 mm, 26**–**28 AWG (40 pcs)**	**1**	**2**	**2**	**Shopee**	**Other**
**CA1**	**Silicone 2-conductor wire, 18 AWG (pump power), 2 *m***	**2**	**1.5**	**3**	**Shopee**	**Other**
**CA2**	**Stranded 3-conductor cable, 22 AWG (sensor loom), 2 *m***	**2**	**1.25**	**2.5**	**Shopee**	**Other**
**SH1**	**Shielded twisted pair, 24 AWG (soil-pH electrode), 2 *m***	**2**	**1.5**	**3**	**Shopee**	**Other**
**CON1**	**JST-XH 2.54 mm housings + crimp terminals (assorted kit)**	**1**	**3.5**	**3.5**	**Shopee**	**Other**
**CON2**	**Screw terminal blocks 3.5 mm, 2-pin & 3-pin (pack)**	**1**	**2**	**2**	**Shopee**	**Other**
**HS1**	**Heat-shrink tubing assortment (1**–**10 mm)**	**1**	**2**	**2**	**Shopee**	**Other**
**CT1**	**Cable ties (nylon), 100 pcs**	**1**	**1**	**1**	**Shopee**	**Other**
**G1**	**Cable glands M12 (pack of 4)**	**1**	**3**	**3**	**Shopee**	**Other**
**JB1**	**Waterproof junction box 4x4 CM**	**1**	**4**	**4**	**Shopee**	**Other**
**PWR1**	**USB adapter 5 V 2 A (alt. to B1 battery set)**	**1**	**5**	**5**	**Shopee**	**Electronic**
**CABL1**	**USB-C to USB-A data cable, 1 m (24/28 AWG)**	**1**	**2.5**	**2.5**	**Shopee**	**Electronic**
**R1**	**2.4 GHz Wi-Fi router (802.11b/g/n) TP Link WR840n**	**1**	**15**	**15**	**Shopee**	**Electronic**
**Grand Total**				**USD 119.00**		

## 4 Bill of materials summary

.

## 5 Build instructions

*The steps below summarize how to assemble a working TerraGrow unit from individual components:*

i. *Print and test-fit the top-cap: ESP32 (U1), DMS/CD4051 boards, relay (K1), battery (B1), and DHT11 vent.*
ii. *Assemble electronics: solder the DMS boards, add headers, and check continuity on the mux and relay lines.*
iii. *Build probes: make three copper soil-moisture pairs and one aluminum pH pair with consistent spacing/depth, then label and insulate.*
iv. *Wire: route CH1–CH3 to DMS-1 and pH to DMS-2, tie both COM lines to one ESP32 ADC pin, share S0–S2, and common ground.*
v. *Mount DHT11 in a shaded vent, wire power/data, connect relay and pump (normally open), and install the battery/regulator.*
vi. *Final assembly: mount all hardware in the top-cap, route S1 (moisture), S3 (pH), and pump leads through glands, seal, and strain-relieve.*
vii. *Flash firmware, set Wi-Fi and fuzzy rules, confirm telemetry and pump control, then calibrate moisture (dry/field-wet) and pH (buffers 4/7/10).*

**Fig. 5:**
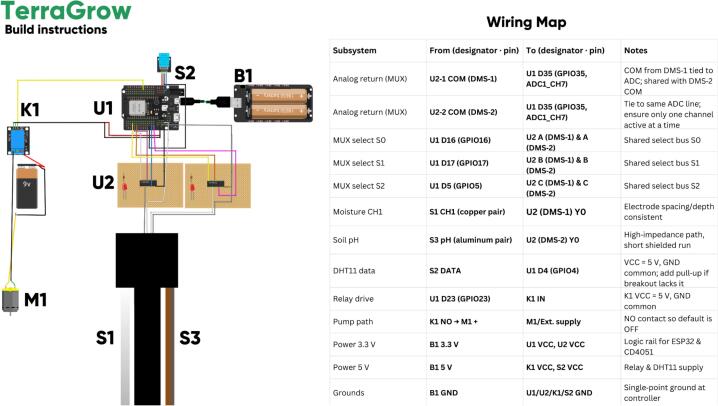
Build instructions. The assembly view shows U1 (ESP32) at the top–cap, the DHT11 (S2) mounted near the vent, the battery/regulator (B1) feeding the 3.3  V/5 V rails, the DMS board (U2, CD4051) centered for short analog runs to the stake harness (S1 = moisture bundle, S3 = pH bundle), and the relay (K1) driving the submersible pump (M1) through the normally–open contact.

Fig. 5 *shows the layout: ESP32 (U1) at the top-cap, DHT11 (S2) near the vent, B1 powering 3.3 V/5 V rails, CD4051 (U2) centered for short analog runs to harnesses (S1 = moisture, S3 = pH), and relay (K1) driving pump M1.*

## 6 Operation instructions

**Fig. 6:**
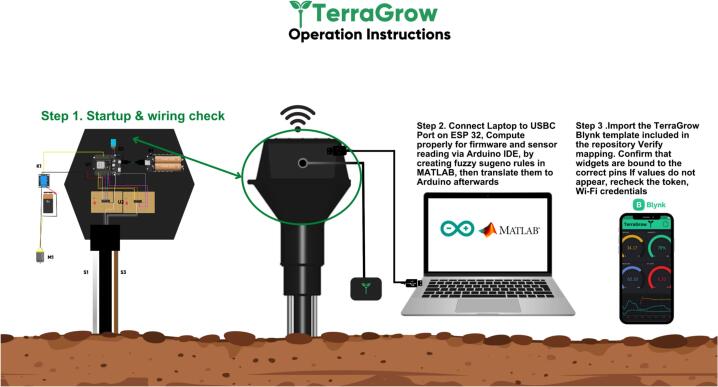
TerraGrow operation instructions.

This section explains how to commission and operate TerraGrow from power-up to autonomous use. Fig. 6 shows the overall workflow in three steps. First check wiring: the ESP32, CD4051 boards, DHT11, and relay–pump wiring must be secure, sensors must be on the correct channels, and 3.3 V / 5 V / GND must all be connected. Connect the ESP32 with USB-C, flash the firmware in the Arduino IDE, and check the serial monitor to confirm moisture, pH, temperature, and humidity values. Set up Blynk by importing the TerraGrow template, adding the device token, and checking that the virtual pins match (V1 pH, V3 moisture, V4 RH, V5 temperature, V6 manual, V7 pump state, V9 auto/manual). If widgets are blank, fix Wi-Fi or the token. Once data is visible, test the pump from the app, then switch V9 to Auto for unattended irrigation.

### 6.1 Startup & wiring check

*Before powering the unit, compare the build to* Fig. 5*, ESP32 (U1) in the top-cap, DMS/CD4051 boards in the center with short analog runs, DHT11 (S2) at the vent, relay (K1) driving the pump (M1) through the normally-open contact, S1 (moisture) and S3 (pH) harnesses routed cleanly, and B1 supplying 3.3 V / 5 V.*

i. *With power off: make sure all grounds are tied together, both DMS COM lines go to the ESP32 ADC pin, S0–S2 from the ESP32 go to both CD4051s, the DHT11 data pin and relay input go to the correct GPIOs, and there are no shorts on 3.3 V / 5 V.*
ii. *Power on with a current-limited supply: check that 3.3 V and 5 V are present, nothing overheats, and the ESP32 boots and prints the pin map / Wi-Fi attempt over serial.*
iii. *Sensor check: moisture reads “dry” in air and drops when briefly bridged; DHT11 temperature/RH looks reasonable; pH is stable.*
iv. *Actuation/dashboard: trigger manual override, confirm the relay clicks and drives the pump line, and verify the app shows live moisture, pH, temperature, humidity, and pump state.*

### 6.2 Data preparation & control algorithm

*The ESP32 cycles through moisture (CH1–CH3), pH, and DHT11 by stepping the CD4051 select lines, sampling the ADC, and repeating on timed intervals (≈5 s moisture, 10 s temp/RH, 15 s pH). Readings are smoothed and checked so only valid data reach the controller and dashboard.*

*Calibration converts ADC codes to engineering units. With a 10-bit ADC the measured voltage* $n\in \left[0,1023\right]$ *is*

(2) $$V_{\text{ADC}}\left(n\right)=\frac{n}{1023}\;V_{\text{ref}}.$$

*After buffer calibration, soil pH is mapped linearly from the ADC code as*

(3) pH=an+b

*Soil-moisture percentage uses piecewise linear interpolation* $\left(nk,mk\right)$ *are*

(4) $$\hat{m}\left(n\right)=m_{k}+\frac{\left(n-n_{k}\right)}{\left(n_{\left(k+1\right)}-n_{k}\right)}\left(m_{\left(k+1\right)}-m_{k}\right)$$

*When memory is tight a two-point alternative with dry and field-wet codes may be used*

(5) $$\hat{m}\left(n\right)=100\%\frac{n_{\mathrm{wet}}-n}{n_{\mathrm{wet}}-n_{\mathrm{dry}}}$$

*Calibrated inputs are mapped to fuzzy sets and evaluated by a Sugeno controller; its weighted output drives {OFF, MID (∼5 s), ON}. Firmware enforces safety limits (run time, rest, daily cap), halts on sensor faults with alarms, and each cycle updates V1 (pH), V3 (moisture), V4 (RH), V5 (temperature), V7 (pump) while logging data for tuning*.

### 6.3 Pairing with Blynk

**Fig. 7:**
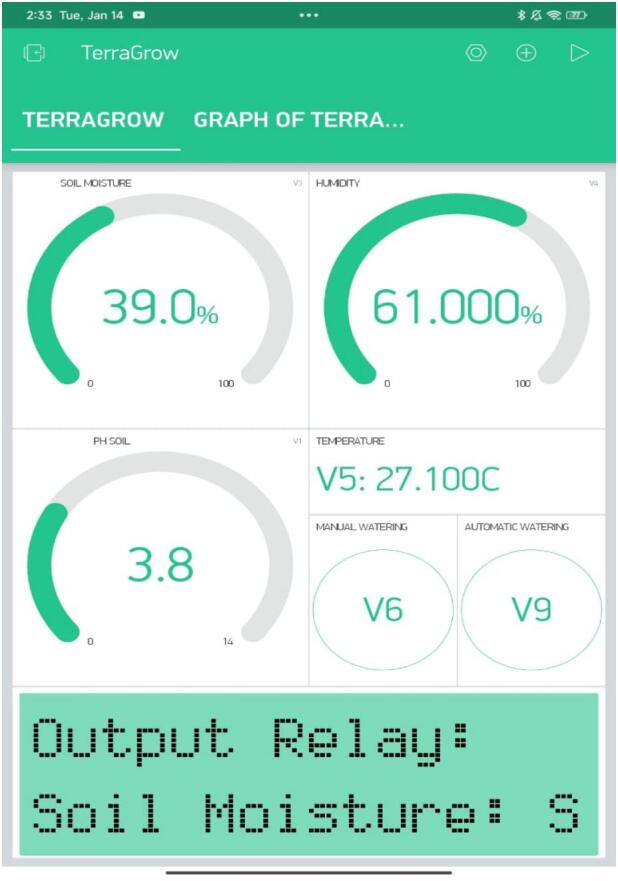
Blynk IoT dashboard configurations.

*Install and sign in to the Blynk app (iOS/Android), import the TerraGrow template (or set up the dashboard using the datastreams in* Table 4*, create a device, copy its Device Token, and add the Wi-Fi details to the firmware. Power the unit and confirm it shows Online. Enable push alerts for low moisture and out-of-range pH, and check that widgets (V1–V9) update. If they don’t, recheck the token, Wi-Fi, and pin mapping. In* Fig. 7 *shows virtual gauges configuration on blynk app.*

**Table 4:** Blynk virtual gauge pairing.

**Signal**	**Virtual Pin**	**Update period**	**Widget type**	**Units**
**Soil moisture CH1**	**V3**	**5 s**	**Time–series chart**	**%**
**Temperature**	**V5**	**10 s**	**Time–series chart**	**°C**
**Relative humidity**	**V4**	**10 s**	**Time–series chart**	**%**
**Soil pH**	**V1**	**15 s**	**Time–series chart**	**pH**
**Pump state**	**V7**	**on–change**	**Indicator**	**—**
**Auto/Manual mode**	**V9**	**on–change**	**Switch**	**—**
**Manual override**	**V6**	**on–press**	**Button (momentary)**	**—**

### 6.4 Firmware flow

**Fig. 8:**
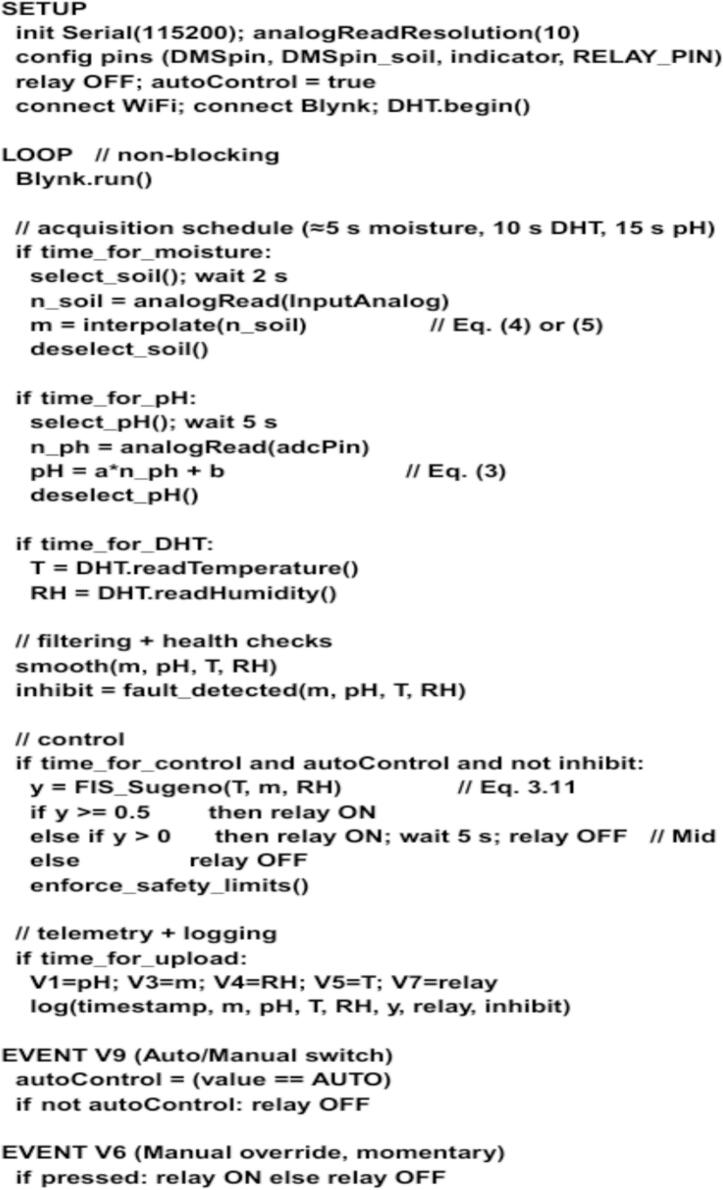
The proposed pseudocode of TerraGrow.

Fig. 8 *summarizes the firmware workflow in compact steps. The setup phase initializes the pins, ADC resolution, Wi-Fi, Blynk, and DHT11 sensor. The main loop runs on a non-blocking schedule, sampling moisture every 5 s, temperature and humidity every 10 s, and pH every 15 s. Each cycle applies filtering and health checks, runs the fuzzy evaluation, enforces safety limits, updates the Blynk dashboard, and logs data with timestamps. Two event handlers complete the loop: the Auto/Manual switch activates or disables the fuzzy controller, and the manual override button directly toggles the relay while preserving safety constraints.*

**Fig. 9:**
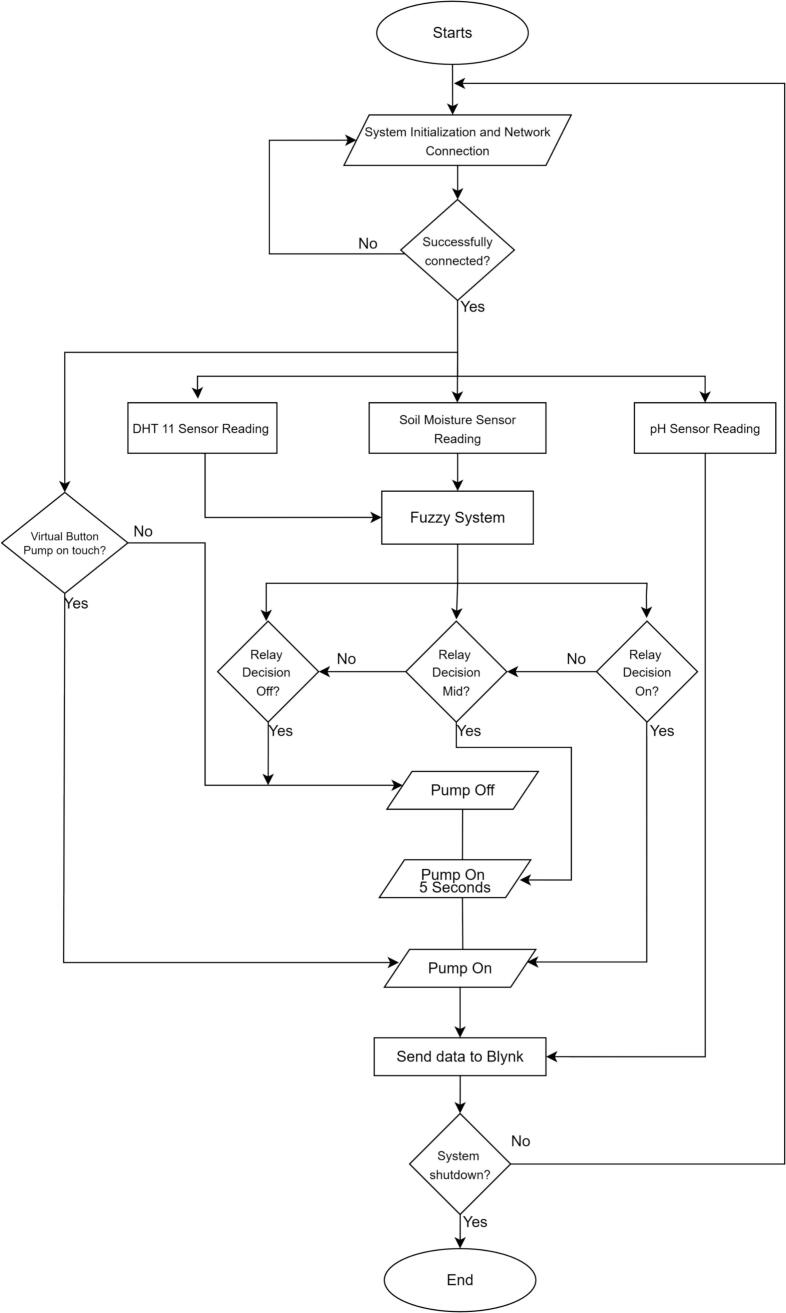
TerraGrow firmware programs flowchart.

Fig. 9 *shows how the firmware runs from start to finish. The ESP32 starts up, connects to Wi-Fi and Blynk, reads all sensors, runs the Sugeno fuzzy logic, and turns the pump relay either Off, Mid (about 5 s), or On. It then sends the readings and pump state to Blynk and loops. The app has a manual button to run the pump briefly, and a switch to choose Auto (fuzzy control) or Manual. If Wi-Fi is not available, the ESP32 keeps trying to connect before it starts normal operation.*

### 6.5 First‑run procedure

**Fig. 10:**
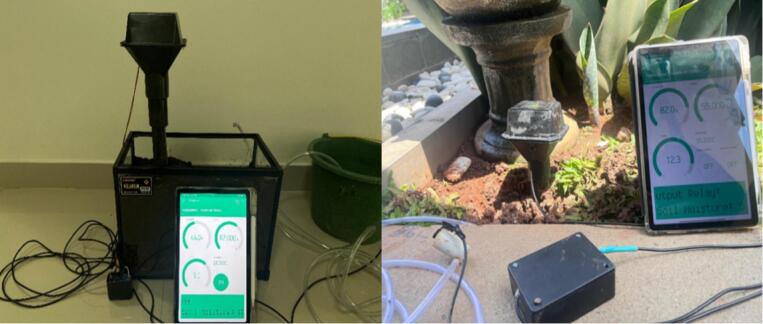
Bench and field first-run setups.

*This section briefly explains how to bring a new TerraGrow unit online. Verify all wiring, power, and sensor readings, then connect the ESP32 to Blynk and ensure telemetry updates correctly. Run one supervised irrigation cycle to confirm the fuzzy controller’s operation.* Fig. 10 *shows a safe bench test with a pot and reservoir, and in the right side is a field setup near plant roots for real-condition verification.*

*Procedure (concise):*

i. *Prepare the site—fill the reservoir, prime the pump, and keep the DHT11 vent shaded.*
ii. *Insert electrodes—place copper (moisture) and aluminum (pH) pairs at target depth with even spacing and strain relief.*
iii. *Power and connect—apply power; wait for the ESP32 to connect to Wi-Fi/Blynk.*
iv. *Check dashboard—verify V3 (moisture), V5 (temp), V4 (RH), V1 (pH), and V7 (pump) show plausible readings.*
v. *Calibrate (optional)—set dry/field-wet points and correct pH offset if needed.*
vi. *Enable Auto mode—toggle V9; dry/hot conditions trigger On/Mid (∼5 s), wet/cool keep Off.*
vii. *Inspect—check for leaks, stable power rails, and isolated wiring.*
viii. *Log and review—run one irrigation cycle and confirm soil moisture stabilizes within the target band*

## 7 Validation and characterization

### 7.1 Data collection and analysis

**Fig. 11:**
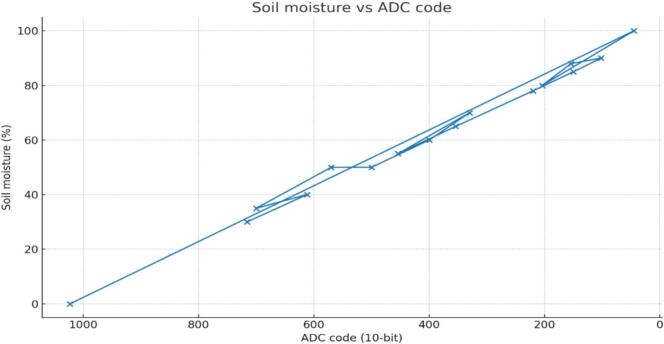
Soil moisture vs ADC.

**Fig. 12:**
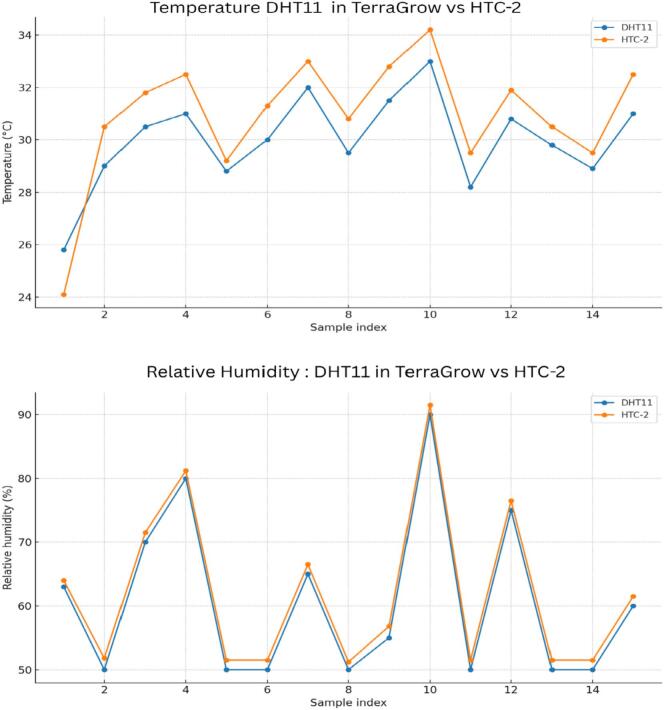
Temperature and Relative humidity DHT11 vs HTC-2.

**Fig. 13:**
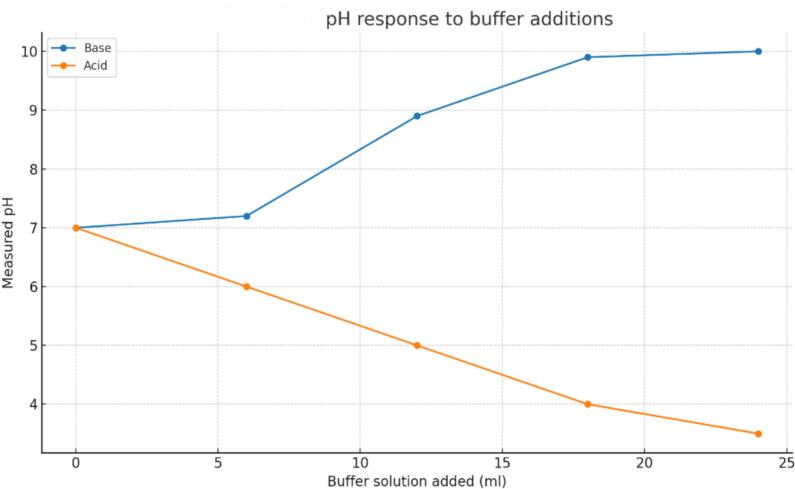
pH response to buffer additions.

*We analyzed four datasets: soil-moisture calibration with copper probes, air temperature–humidity comparison (DHT11* vs*. HTC-2), soil-pH trials using aluminum electrodes with controlled acid/alkali additions, and relay responses under typical environmental scenarios. Raw ADC readings were converted to engineering units using calibration maps, with results summarized in* Table 5, Table 6, Table 7, Table 8 *and* Fig. 11, Fig. 12, Fig. 13*. For soil moisture,*

**Table 5:** Soil-moisture: ADC vs Percentage %.

***No Sample***	***ADC Value***	***Moisture(%)***	***Moisture Predicted (%)***	***Δ(%)***
*1*	*716*	*30*	*30.77*	*−0.77*
*2*	*612*	*40*	*40.99*	*−0.99*
*3*	*700*	*35*	*32.34*	*2.66*
*4*	*570*	*50*	*45.12*	*4.88*
*5*	*500*	*50*	*52*	*−2*
*6*	*400*	*60*	*61.82*	*−1.82*
*7*	*330*	*70*	*68.7*	*1.3*
*8*	*454*	*55*	*56.52*	*−1.52*
*9*	*354*	*65*	*66.34*	*−1.34*
*10*	*220*	*78*	*79.51*	*−1.51*
*11*	*150*	*85*	*86.39*	*−1.39*
*12*	*102*	*90*	*91.11*	*−1.11*
*13*	*154*	*88*	*86*	*2*
*14*	*204*	*80*	*81.09*	*−1.09*
*15*	*45*	*100*	*96.71*	*3.29*
*16*	*1023*	*0*	*0.6*	*−0.6*

**Table 6:** DHT11 vs HTC-2.

***No Sample***	**DHT11 RH (%)**	**DHT11 Temp (C)**	**HTC2 RH (%)**	**HTC2 Temp (C)**	***Δ* RH (%)**	***Δ*Temp (C)**
*1*	*63*	*25.8*	*64*	*24.1*	*1*	*−1.7*
*2*	*50*	*29*	*51.8*	*30.5*	*1.8*	*1.5*
*3*	*70*	*30.5*	*71.5*	*31.8*	*1.5*	*1.3*
*4*	*80*	*31*	*81.2*	*32.5*	*1.2*	*1.5*
*5*	*50*	*28.8*	*51.5*	*29.2*	*1.5*	*0.4*
*6*	*50*	*30*	*51.5*	*31.3*	*1.5*	*1.3*
*7*	*65*	*32*	*66.5*	*33*	*1.5*	*1*
*8*	*50*	*29.5*	*51.2*	*30.8*	*1.2*	*1.3*
*9*	*55*	*31.5*	*56.8*	*32.8*	*1.8*	*1.3*
*10*	*90*	*33*	*91.5*	*34.2*	*1.5*	*1.2*
*11*	*50*	*28.2*	*51.5*	*29.5*	*1.5*	*1.3*
*12*	*75*	*30.8*	*76.5*	*31.9*	*1.5*	*1.1*
*13*	*50*	*29.8*	*51.5*	*30.5*	*1.5*	*0.7*
*14*	*50*	*28.9*	*51.5*	*29.5*	*1.5*	*0.6*
*15*	*60*	*31*	*61.5*	*32.5*	*1.5*	*1.5*

**Table 7:** Soil pH tests.

***Condition***	***Buffer_ml***	***ADC_code***	***Measured_pH***
*Base*	*0*	*272*	*7*
*Base*	*6*	*232*	*7.2*
*Base*	*12*	*161*	*8.9*
*Base*	*18*	*120*	*9.9*
*Base*	*24*	*116*	*10*
*Acid*	*0*	*266*	*7*
*Acid*	*6*	*295*	*6*
*Acid*	*12*	*338*	*5*
*Acid*	*18*	*351*	*4*
*Acid*	*24*	*383*	*3.5*

**Table 8:** Automated watering of relay summary.

***No***	***ResponseTime_s***	***TempStatus***	***SoilMoistureStatus***	***AirHumidityStatus***	***RelayOutput***
*1*	*5*	*Cold*	*Low*	*Dry*	*On*
*2*	*4*	*Hot*	*High*	*Normal*	*Off*
*3*	*3*	*Good*	*Medium*	*Moist*	*Mid*
*4*	*4*	*Cold*	*Medium*	*Normal*	*On*
*5*	*5*	*Hot*	*Low*	*Dry*	*Off*
*6*	*4*	*Good*	*Low*	*Normal*	*On*
*7*	*5*	*Cold*	*Low*	*Moist*	*Off*
*8*	*2*	*Hot*	*Medium*	*Dry*	*On*
*9*	*6*	*Good*	*High*	*Moist*	*Mid*
*10*	*4*	*Cold*	*High*	*Normal*	*Off*

16 Paired samples (10-bit ADC vs. % moisture) showed the expected inverse trend—higher moisture produced lower ADC values. A least-squares fit yielded R^2^ ≈ 0.994 and r ≈ −0.997 over ADC 45–1023 (0–100 % moisture), indicating strong linearity. As shown in Fig. 11, a 100-code ADC drop corresponds to about a 9.8 % increase in moisture, confirming accurate two-point (dry/field-wet) calibration

*From 15 paired readings, the HTC-2 measured slightly higher than the DHT11 by + 1.47 % RH (SD 0.21 %, RMSE 1.48 %) and + 0.95 °C (SD 0.81 °C, RMSE 1.23 °C) with strong correlations (r ≈ 0.9999 for RH; r ≈ 0.969 for temperature). These small, consistent offsets are typical for low-cost sensors and do not affect the fuzzy LOW/MEDIUM/HIGH classifications. If needed, a single-point offset can correct the difference.* Table 6 *lists the paired readings and deltas, while* Fig. 12 *shows both sequences closely aligned.*

*Two five-point tests were performed with incremental base and acid additions (0–24 mL). After buffer calibration, the alkaline series showed a slope of + 0.145 pH/mL (R2 ≈ 0.916) and the acidic series − 0.150 pH/mL (R2 ≈ 0.988), covering* pH *3.5–10.0. When combined, regression of* pH *versus ADC yielded R2 ≈ 0.978 with a negative slope—higher ADC at lower pH—consistent with the firmware’s linear mapping.* Table 7 *summarizes both datasets, showing smooth, monotonic responses, while* Fig. 13 *plots pH versus solution volume, illustrating clear separation between the acid and base trends.*

*Ten scenarios combining temperature, soil-moisture, and air-humidity statuses produce a balanced distribution (On: 4, Off: 4, Mid: 2), matching the intended rule base (dry/hot = On; wet/cool = Off; intermediate = Mid). Mean actuation latency—from decision to stable relay state—is 4.2 s overall, with class-wise means of On ≈ 3.75 s, Off ≈ 4.50 s, and Mid ≈ 4.50 s (the Mid class includes a deliberate ∼ 5 s pulse).*Table 8 *lists representative scenarios (temperature status, soil-moisture status, and air-humidity status) and the resulting relay class selected by the Sugeno controller (Off, Mid, On).*

*Across all datasets, the results confirm reliable system performance: strong inverse correlation for moisture, high cross-sensor agreement between DHT11 and HTC-2 with minimal offsets, a stable linear pH–ADC response after calibration, and consistent low-latency relay actions. The main variability arises from soil conductivity and ionic strength, which the two-point calibration in Eq.* (4) *and Eq.* (5) *effectively compensates for, while the pH fit in Eq.* (3) *can be updated if electrode or soil chemistry changes* [35]*.*

### 7.2 Discussion

*TerraGrow provides automated irrigation with consistent control and measurable reductions in unnecessary watering. Many low-cost IoT or fuzzy-based systems can switch a pump from a soil-moisture threshold, but they usually use only one probe, do not compensate for temperature and humidity, do not monitor soil chemistry, and often do not report whether soil moisture actually stayed inside a defined operating band over time* [[19], [20], [21]]*. In TerraGrow, three soil-moisture channels in the root zone are calibrated in the field (dry and field-wet) and then sent to the fuzzy controller as engineering values instead of raw ADC codes. With this calibration in place, the system was able to keep moisture in the intended 60–80 % band during greenhouse and pot-soil runs, which shows that the sensing chain and the rule base work together to deliver repeatable, bounded irrigation rather than wide, undocumented swings* [20,26]*. This is a clear control-accuracy outcome that many single-probe on/off designs do not document, and it makes TerraGrow quantitatively stronger because it reports the operating band, not only that the pump can be turned on. The calibration dataset also showed a strong, nearly linear inverse trend between ADC and percent moisture, so small real changes in soil water were visible to the controller and could be acted on* [25]*. Because the fuzzy rules operate on calibrated values, the controller can tell the difference between truly dry and moderately dry and can issue different actions such as off, short pulse, or full run. This finer actuation is what cuts superfluous irrigation, since the system does not need to run a long pump cycle when the soil is already near the upper edge of the 60–80 % window. This accuracy mainly comes from the way the system is designed and built, with the sensing stack arranged cleanly in the top-cap, analog runs kept short through the DMS, and the ESP32 executing the Sugeno rules locally so the sensors deliver stable values and the controller can translate them into the correct pump time.*

*TerraGrow also improves decision relevance by using microclimate inputs. Air temperature and relative humidity are read and used to modulate watering, so hot and dry conditions can promote irrigation while cool and humid conditions can suppress it even when soil moisture is trending low* [27]*. Many earlier systems trigger only on soil moisture, which can lead to over-watering on cloudy or humid days. In TerraGrow, graded outputs follow environmental demand, so water use is reduced for a real and measurable reason, not only because of a tighter threshold* [31]*. TerraGrow also logs soil pH in situ using a buffer-calibrated aluminum electrode and shows it alongside moisture and humidity. This makes it possible to spot pH shifts after fertilization or liming without extra instruments, something that most comparable low-cost irrigation nodes do not support* [22,27,29,32,34]*.*

*Taken together, these results show that TerraGrow keeps soil moisture in a defined target band, reduces unnecessary irrigation through short pulses near the upper limit, and drives the pump with predictable timing and safety limits using only low-cost sensors and a single ESP32* [22,34]*. Compared with earlier low-cost builds that only demonstrate basic pump triggering, this provides an explicit operating window, calibrated sensing, and multi-sensor–aware irrigation decisions* [19,20,21,27,31]*.*

### 7.3 Limitations and Future work

*TerraGrow has been tested in small crops and small greenhouse setups, not yet in long-term, multi-zone, or hectare-scale trials. Next steps include seasonal durability tests, adding higher-stability sensors, and extra inputs such as rain detection, on-site weather sensing, and basic forecast data to further reduce unnecessary irrigation* [24,28]*. The current design controls one local zone with a single ESP32 and Wi-Fi/Blynk, which suits small growers but is not yet optimized for orchards or multi-plot layouts; multi-node scaling with LoRa-type field nodes reporting to one gateway will allow several pumps and valves to be coordinated across a site* [21,33]*. AI integration for precision agriculture is also planned by using fuzzy logic as a simple, explainable form of AI on device. Fuzzy theory can encode expert rules and handle uncertainty in moisture, temperature, humidity, and pH, making it a practical bridge to more advanced analytics on the edge or cloud* [36,37]*. This roadmap includes predictive irrigation and fertigation recommendations, automatic liquid-fertilizer dosing via added nutrient sensors (EC, ion-selective, or NPK probes) and an auxiliary dosing path, and the use of weather inputs to adapt set-points and skip evaporation low or rainy days, improving water and input efficiency while preserving low cost and ease of use* [24,29]*.*

## CRediT authorship contribution statement

**Prima Wijayakusuma:** Writing – review & editing, Writing – original draft, Software. **Galang Persada Nurani Hakim:** Validation, Software, Methodology, Formal analysis, Data curation, Conceptualization. **Bin Li:** Writing – review & editing, Supervision.

## Declaration of competing interest

*The authors declare that they have no known competing financial interests or personal relationships that could have appeared to influence the work reported in this paper.*
